# Supplementary material for: Diagnostic accuracy of S-Detect in distinguishing benign and malignant thyroid nodules: A meta-analysis
Source: PLoS One. 2022 Aug 5;17(8):e0272149. doi: 10.1371/journal.pone.0272149 (PMC9355179; doi:10.1371/journal.pone.0272149)
Supplement: S1 Checklist — (DOC) [file pone.0272149.s001.doc]

| **Section/topic** | **#** | **Checklist item** | **Reported on page #** |
| --- | --- | --- | --- |
| **TITLE** | | |  |
| Title | 1 | Diagnostic accuracy of S-Detect in distinguishing benign and malignant thyroid nodules: a meta-analysis | 1 |
| **ABSTRACT** | | |  |
| Structured summary | 2 | Objectives: This meta-analysis aimed to identify the diagnostic accuracy of S-Detect in distinguishing benign and malignant thyroid nodules.  Methods: We searched PubMed, Cochrane Library, and CBM databases from inception to August 1st 2021. Meta-analysis was conducted using STATA version 14.0 and Meta-Disc version 1.4 softwares. We calculated the summary statistics for sensitivity (Sen), specificity (Spe), positive and negative likelihood ratio (LR+/LR−), diagnostic odds ratio(DOR), and receiver operating characteristic (SROC) curve. The Cochran's Q-statistic and I2 test were used to evaluate potential heterogeneity between studies. To evaluate the influence of single studies on the overall estimate, a sensitivity analysis was performed. We also performed meta-regression analyses to investigate potential sources of heterogeneity.  Results: Seventeen studies that met all inclusion criteria were included in this meta-analysis. A total of 1118 thyroid malignant nodules and 1595 thyroid benign nodules were assessed. All thyroid nodules were histologically confirmed after S-Detect. The pooled Sen was 0.87 (95%CI=0.84-0.89); the pooled Spe was 0.74 (95%CI=0.66-0.81). The pooled LR+ was 3.37 (95%CI=2.53-4.50); the pooled negative LR−was 0.18 (95%CI=0.15-0.21). The pooled DOR of S-Detect in the diagnosis of thyroid nodules was 18.83 (95% CI=13.21-26.84). The area under the SROC curve was 0.89 (SE=0.0124). We found no evidence for publication bias (t=0.25, P=0.80).  Conclusions: Our meta-analysis indicates that S-Detect may have high diagnostic accuracy in distinguishing benign and malignant thyroid nodules. | 2 |
| **INTRODUCTION** | | |  |
| Rationale | 3 | In recent years, the incidence rate of thyroid diseases has been increasing year by year, which is closely related to the progress of biological characteristics and ultrasound diagnosis technology.[1] Ultrasonography has the advantage of high sensitivity in the diagnosis of thyroid nodule, and is is the first choice for clinical diagnosis and differentiation of thyroid cancer.[2] However, the diagnostic accuracy of this technique is often related to the level of doctors' experience. Computer aided diagnosis (CAD) technology is one of the hotspots of artificial intelligence and modern medical research.[3] Ultrasonic S-Detect(Samsung Medison Co. Ltd., Seoul, South Korea) technology is a computer-aided diagnosis technology, which uses convolution neural network deep learning algorithm to evaluate thyroid nodules according to Ti-RADS dictionary. The deep learning model is used to automatically detect and analyze the boundary, shape, internal echo and other information of nodules, overcome the interference of human factors, and objectively judge the benign and malignant nodules.[4] Previous studies have shown that S-Detect technology has a high accuracy in the differential diagnosis of thyroid nodules. [5-7] However, the results of these studies have been contradictory and the sample sizes were not enough. | 2 |
| Objectives | 4 | Therefore, the present meta-analysis aimed at determining the accuracy of S-Detect for the differential diagnosis of benign and malignant thyroid nodules. | 2 |
| **METHODS** | | |  |
| Protocol and registration | 5 | N/A. |  |
| Eligibility criteria | 6 | The following 4 criteria were required for each study: (1) the study design must be a clinical cohort study or diagnostic test, (2) the study must relate to the accuracy of S-Detect for the differential diagnosis of benign and malignant thyroid nodules, (3) all thyroid nodules were histologitally confirmed, and(4) published data in the fourfold (2×2) tables must be sufficient. If the study did not meet all of these inclusion criteria, it was excluded. The most recent publication or the publication with the largest sample size was included when the authors published several studies using the same subjects. | 2 |
| Information sources | 7 | We searched PubMed, Cochrane Library, and CBM databases without language restrictions.We also performed a manual search to find other potential articles. | 2 |
| Search | 8 | The following keywords and MeSH terms were used: ["thyroid cancer" or "thyroid neoplasm" or "thyroid tumor" or "thyroid nodule "] and [“S-Detect” or “smart detect” or “artificial Intelligence” or “computer aid diagnosis” or “machine intelligent”]. We also performed a manual search to find other potential articles. | 2 |
| Study selection | 9 | Initially, the searched keywords identified 40 articles. We reviewed the titles and abstracts of all articles and excluded 17 articles; full texts and data integrity were also reviewed and 6 were further excluded. Finally, 17 studies that met all inclusion criteria were included in this meta-analysis | 3 |
| Data collection process | 10 | Relevant data were systematically extracted from all included studies by two researchers using a standardized form. | 3 |
| Data items | 11 | The researchers collected the following data: the first author's surname, publication year, language of publication, study design, sample size, number of lesions, source of the subjects, "gold standard," and diagnostic accuracy. The true positives (TP), true negatives (TN), false positives (FP), and false negatives (FN) in the fourfold (2 x 2) tables were also collected. | 3 |
| Risk of bias in individual studies | 12 | Methodological quality was independently assessed by two researchers based on the quality assessment of studies of diagnostic accuracy studies (QUADAS) tool. | 3 |
| Summary measures | 13 | The QUADAS criteria included 14 assessment items. Each of these items was scored as "yes" (2), "no" (0), or "unclear"(1). The QUADAS score ranged from 0 to 28, and a score≧22 indicated good quality. | 3 |
| Synthesis of results | 14 | The STATA version 15.1 software was used for Meta-analysis. We calculated the pooled summary OR and its 95% confidence interval(CI). The Cochran’s Q-statistic and I^2^ test were used to evaluate potential heterogeneity between studies. If Q test shows a P<0.05 or I^2^ test exhibits>50% which indicates significant heterogeneity, the random-effect model was conducted, or else the fixed-effects model was used. | 3 |

Page 1 of 2

| **Section/topic** | **#** | **Checklist item** | **Reported on page #** |
| --- | --- | --- | --- |
| Risk of bias across studies | 15 | We conducted Begger’s funnel plots and Egger’s linear regression test to investigate publication bias. | 3 |
| Additional analyses | 16 | In order to evaluate the influence of single study on the overall estimate, sensitivity analysis was performed. | 3 |
| **RESULTS** | | |  |
| Study selection | 17 | 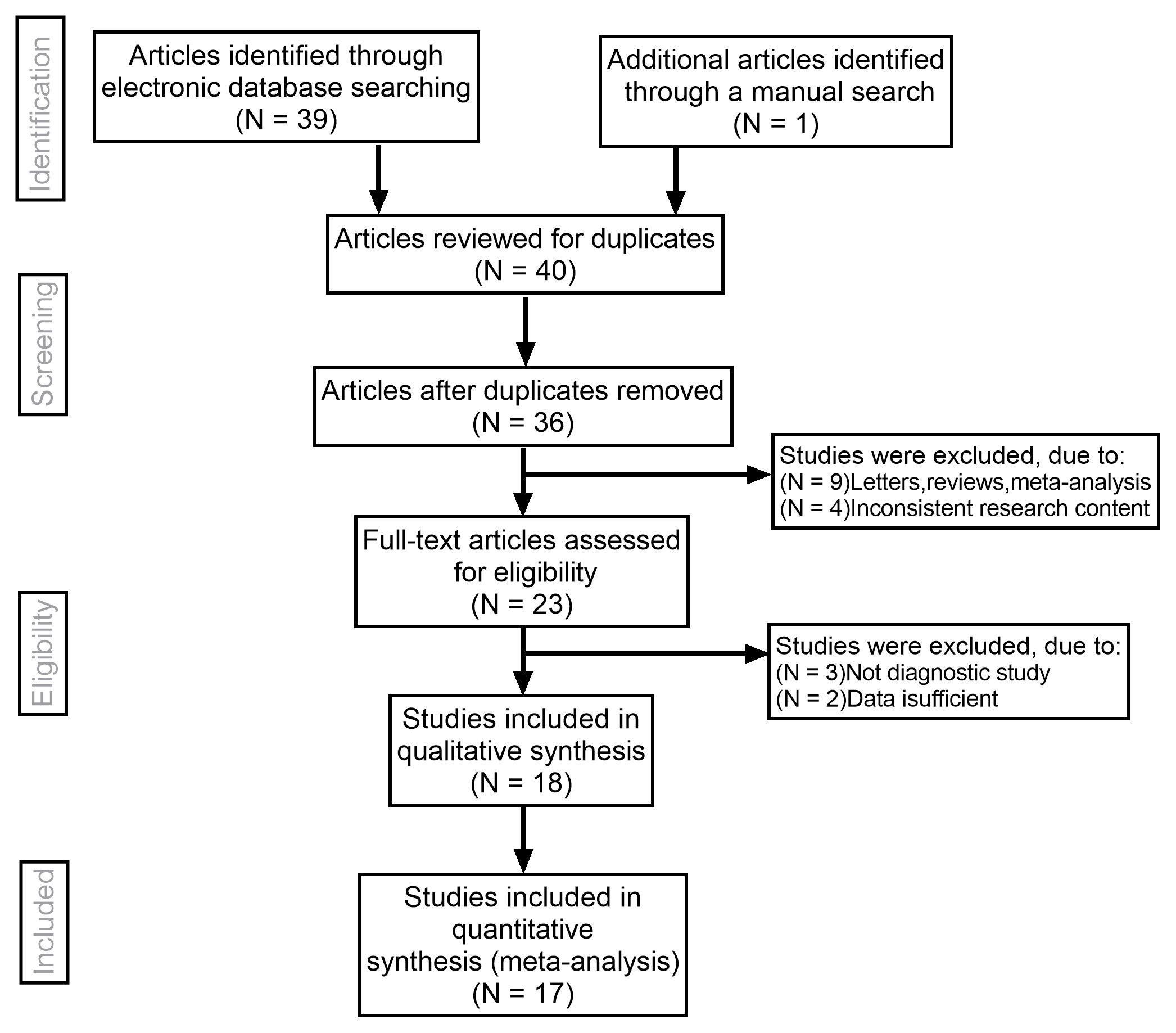 | 3 |
| Study characteristics | 18 | The QUADAS scores of all included studies were ≥24. | 3 |
| Risk of bias within studies | 19 | | Table 1 Baseline characteristics and methodological quality of all included studies | | | | | | | | | | | | | --- | --- | --- | --- | --- | --- | --- | --- | --- | --- | --- | --- | | First author | Year | Country | Language | Sample size | Age(Years) | Instrument | S-Detect 2×2 table | | | | QUADAS score | | TP | FP | FN | TN | | Xing BY [9] | 2021 | China | Chinese | 152 | 46.5±12.8 | Samsung RS80A | 80 | 9 | 12 | 51 | 24 | | Bi Y [10] | 2021 | China | Chinese | 125 | 46.2±11.5 | Samsung RS80A | 62 | 7 | 16 | 40 | 25 | | LI Q [11] | 2021 | China | Chinese | 183 | 21-67 | Samsung RS80A | 103 | 19 | 12 | 49 | 26 | | Fang MD [12] | 2021 | China | Chinese | 94 | 44.9±11.8 | Samsung RS80A | 55 | 7 | 2 | 30 | 25 | | Chen C [13] | 2020 | China | Chinese | 136 | 47.5±15.4 | Samsung RS80A | 49 | 40 | 7 | 40 | 24 | | Han H [14] | 2018 | China | Chinese | 93 | 45.4±12.5 | Samsung RS80A | 39 | 28 | 5 | 21 | 25 | | Szczepanek-Parulska E [15] | 2020 | Poland | English | 133 | 49.5±15.5 | Samsung RS80A | 59 | 13 | 7 | 54 | 26 | | Wei Q [16] | 2020 | China | English | 204 | 46±12 | Samsung RS80A | 84 | 39 | 8 | 73 | 25 | | Barczyński M [17] | 2020 | Poland | English | 50 | 47.5±15.0 | Samsung RS85A | 9 | 8 | 1 | 32 | 25 | | Kim HL [18] | 2019 | Korea | English | 218 | 22-81 | Samsung RS80A | 69 | 23 | 17 | 109 | 27 | | Xia SJ [19] | 2019 | China | English | 180 | 21-83 | Samsung RS80A | 86 | 50 | 9 | 35 | 26 | | Choi YJ [4] | 2018 | Korea | English | 102 | 45（25-76） | Samsung RS80A | 39 | 15 | 4 | 44 | 26 | | Han M [5] | 2020 | Korea | English | 454 | 49（8-81） | Samsung RS85A | 136 | 52 | 31 | 235 | 26 | | Yoo YJ [6] | 2018 | Korea | English | 117 | 43（22-81） | Samsung RS80A | 40 | 8 | 10 | 59 | 24 | | Chung SR [7] | 2020 | Korea | English | 165 | 51(22-94) | Samsung RS80A | 23 | 17 | 2 | 123 | 26 | | Molnár K [20] | 2020 | Hungary | English | 200 | 54(12-88) | Samsung RS85A | 12 | 110 | 3 | 75 | 26 | | Fresilli D [21] | 2020 | Italy | English | 107 | 55 | Samsung RS80A | 19 | 10 | 8 | 70 | 25 | | TP true positive, TN true negative, FP false positive, FN false negative, QUADAS the quality assessment of studies of diagnostic accuracy studies | | | | | | | | | | | | | 3 |
| Results of individual studies | 20 | 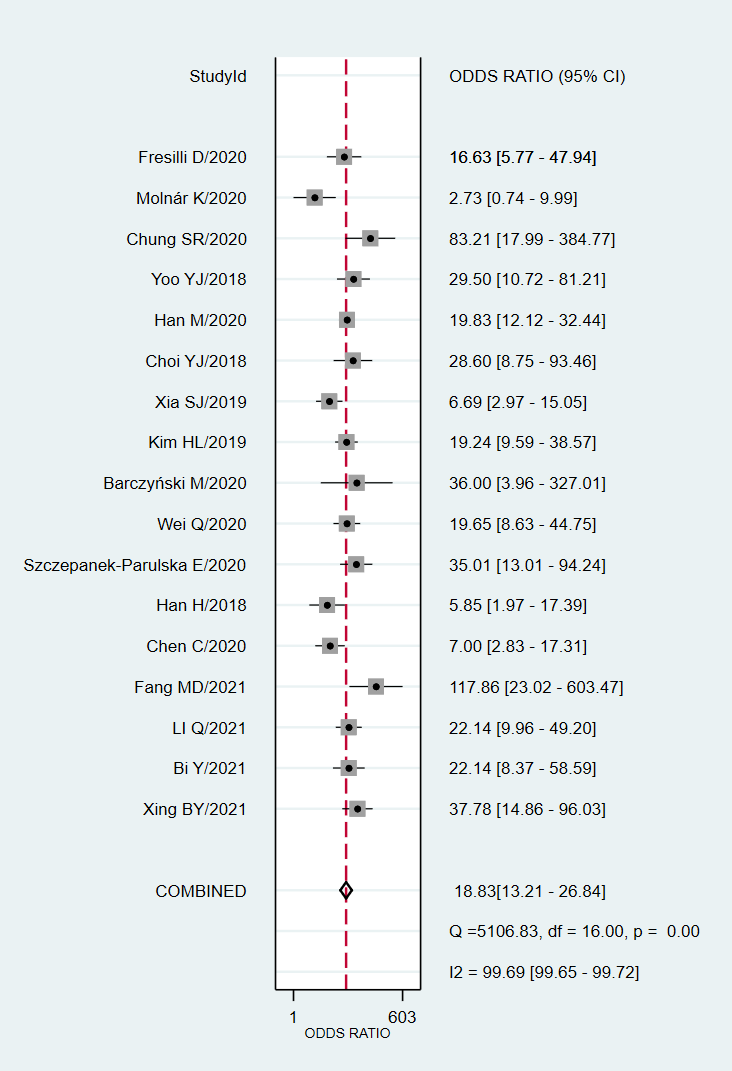 | 3 |
| Synthesis of results | 21 | The pooled Sen was 0.87 (95%CI=0.84-0.89); the pooled Spe was 0.74 (95%CI=0.66-0.81)(Figure 3). There was no significant correlation (r=0.289, P=0.260) between sensitivity and specificity, which indicated that there is no threshold effect. The pooled LR+ was 3.37 (95%CI=2.53-4.50); the pooled negative LR−was 0.18 (95%CI=0.15-0.21)(Figure 4). The pooled DOR of S-Detect in the diagnosis of thyroid nodules was 18.83 (95% CI=13.21-26.84)(Figure 5). The area under the SROC curve was 0.89 (SE=0.0124)(Figure 6). | 3 |
| Risk of bias across studies | 22 | We found no evidence for publication bias(Figure 7). Egger’s test also did not display strong statistical evidence for publication bias (t=0.25, P=0.80). | 3 |
| Additional analysis | 23 | Sensitivity analysis was carried out, and none of them caused obvious interference to the results. | 3 |
| **DISCUSSION** | | |  |
| Summary of evidence | 24 | In conclusion, our meta-analysis suggests that S-Detect may have high diagnostic accuracy in distinguishing benign and malignant thyroid nodules. It can be used as a useful supplement to ordinary ultrasonography. | 3 |
| Limitations | 25 | Despite the demonstrated diagnostic accuracy of S-Detect for thyroid nodule, our study has certain limitations. First, owing to the relatively small sample sizes and low level of quality of the included studies, there were insufficient data to assess the accuracy of SMI. Moreover, the retrospective nature of a meta-analysis can lead to subject selection bias. Importantly, the majority of included studies originated from Asia, which may adversely affect the reliability and validity of our results. | 3 |
| Conclusions | 26 | our meta-analysis suggests that S-Detect may have high diagnostic accuracy in distinguishing benign and malignant thyroid nodules. | 5 |
| **FUNDING** | | |  |
| Funding | 27 | N/A |  |

*From:*  Moher D, Liberati A, Tetzlaff J, Altman DG, The PRISMA Group (2009). Preferred Reporting Items for Systematic Reviews and Meta-Analyses: The PRISMA Statement. PLoS Med 6(7): e1000097. doi:10.1371/journal.pmed1000097

For more information, visit: **www.prisma-statement.org**.

Page 2 of 2
