# Supplementary material for: Diagnostic accuracy of S-Detect in distinguishing benign and malignant thyroid nodules: A meta-analysis
Source: PLoS One. 2022 Aug 5;17(8):e0272149. doi: 10.1371/journal.pone.0272149 (PMC9355179; doi:10.1371/journal.pone.0272149)
Supplement: S1 Table — (DOCX) [file pone.0272149.s002.docx]

| Table 1 Baseline characteristics and methodological quality of all included studies | | | | | | | | | | | |
| --- | --- | --- | --- | --- | --- | --- | --- | --- | --- | --- | --- |
| First author | Year | Country | Language | Sample size | Age(Years) | Instrument | S-Detect 2×2 table | | | | QUADAS score |
|  |  |  |  |  |  |  | TP | FP | FN | TN |  |
| Xing BY [9] | 2021 | China | Chinese | 152 | 46.5±12.8 | Samsung RS80A | 80 | 9 | 12 | 51 | 24 |
| Bi Y [10] | 2021 | China | Chinese | 125 | 46.2±11.5 | Samsung RS80A | 62 | 7 | 16 | 40 | 25 |
| LI Q [11] | 2021 | China | Chinese | 183 | 21-67 | Samsung RS80A | 103 | 19 | 12 | 49 | 26 |
| Fang MD [12] | 2021 | China | Chinese | 94 | 44.9±11.8 | Samsung RS80A | 55 | 7 | 2 | 30 | 25 |
| Chen C [13] | 2020 | China | Chinese | 136 | 47.5±15.4 | Samsung RS80A | 49 | 40 | 7 | 40 | 24 |
| Han H [14] | 2018 | China | Chinese | 93 | 45.4±12.5 | Samsung RS80A | 39 | 28 | 5 | 21 | 25 |
| Szczepanek-Parulska E [15] | 2020 | Poland | English | 133 | 49.5±15.5 | Samsung RS80A | 59 | 13 | 7 | 54 | 26 |
| Wei Q [16] | 2020 | China | English | 204 | 46±12 | Samsung RS80A | 84 | 39 | 8 | 73 | 25 |
| Barczyński M [17] | 2020 | Poland | English | 50 | 47.5±15.0 | Samsung RS85A | 9 | 8 | 1 | 32 | 25 |
| Kim HL [18] | 2019 | Korea | English | 218 | 22-81 | Samsung RS80A | 69 | 23 | 17 | 109 | 27 |
| Xia SJ [19] | 2019 | China | English | 180 | 21-83 | Samsung RS80A | 86 | 50 | 9 | 35 | 26 |
| Choi YJ [4] | 2018 | Korea | English | 102 | 45（25-76） | Samsung RS80A | 39 | 15 | 4 | 44 | 26 |
| Han M [5] | 2020 | Korea | English | 454 | 49（8-81） | Samsung RS85A | 136 | 52 | 31 | 235 | 26 |
| Yoo YJ [6] | 2018 | Korea | English | 117 | 43（22-81） | Samsung RS80A | 40 | 8 | 10 | 59 | 24 |
| Chung SR [7] | 2020 | Korea | English | 165 | 51(22-94) | Samsung RS80A | 23 | 17 | 2 | 123 | 26 |
| Molnár K [20] | 2020 | Hungary | English | 200 | 54(12-88) | Samsung RS85A | 12 | 110 | 3 | 75 | 26 |
| Fresilli D [21] | 2020 | Italy | English | 107 | 55 | Samsung RS80A | 19 | 10 | 8 | 70 | 25 |
| TP true positive, TN true negative, FP false positive, FN false negative, QUADAS the quality assessment of studies of diagnostic accuracy studies | | | | | | | | | | | |
