# Supplementary material for: Diagnostic accuracy of S-Detect in distinguishing benign and malignant thyroid nodules: A meta-analysis
Source: PLoS One. 2022 Aug 5;17(8):e0272149. doi: 10.1371/journal.pone.0272149 (PMC9355179; doi:10.1371/journal.pone.0272149)
Supplement: S2 Table — (DOCX) [file pone.0272149.s003.docx]

| Table 2 Meta-regression analyses of potential source of heterogeneity | | | | | | |
| --- | --- | --- | --- | --- | --- | --- |
| Heterogeneity factors | Coefficient | SE | *P* value | RDOR | 95% CI | |
|  |  |  |  |  | UL | LL |
| Publication year | 0.286 | 0.1938 | 0.1652 | 1.33 | 0.87 | 2.03 |
| Language | 0.315 | 0.4224 | 0.4701 | 1.37 | 0.55 | 3.44 |
| Instrument | 0.747 | 0.4622 | 0.1321 | 2.11 | 0.77 | 5.78 |

SE standard error, RDOR relative diagnostic odds ratio, 95% CI 95 % confidence interval, UL upper limit, LL lower limit
