# Supplementary material for: Diagnostic accuracy of S-Detect in distinguishing benign and malignant thyroid nodules: A meta-analysis
Source: PLoS One. 2022 Aug 5;17(8):e0272149. doi: 10.1371/journal.pone.0272149 (PMC9355179; doi:10.1371/journal.pone.0272149)
Supplement: S1 File — (DOCX) [file pone.0272149.s004.docx]

**Pubmed:** Search: ((((computer aid diagnosis[Title/Abstract]) OR (artificial Intelligence[Title/Abstract])) OR (smart detect[Title/Abstract])) OR (S-Detect[Title/Abstract])) AND ((((thyroid nodule[Title/Abstract]) OR (thyroid tumor[Title/Abstract])) OR (thyroid neoplasm[Title/Abstract])) OR (thyroid cancer[Title/Abstract]))  Filters: Publication date from inception to 2021/08/01.

**Cochrane Library:**


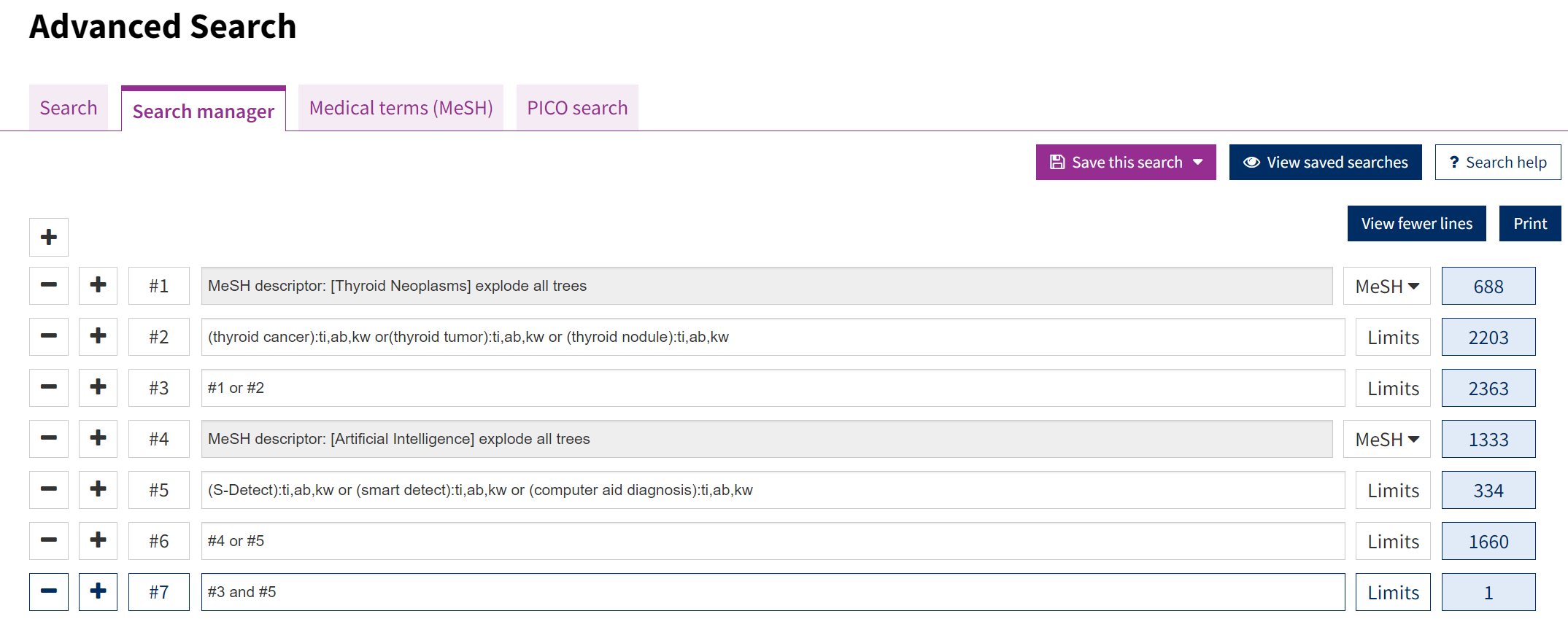


CBM检索式：

| 序号 | 检索表达式 | 结果 | 时间 |
| --- | --- | --- | --- |
| 10 | ((#9)) AND 2015-2021[日期] | 28 | 10:57:24 |
| 9 | (#5) AND (#8) | [49](http://220.166.97.72:9039/zh/javascript:historyLink('(#12) AND (#8) AND (#5)')) | 10:54:50 |
| 8（干预方法） | (#7) OR (#6) | 179232 | 10:48:20 |
| 7 | "S-detect"[不加权:扩展] | 80 | 10:47:20 |
| 6 | "人工智能"[不加权:扩展] | 179163 | 10:46:48 |
| 5(疾病类型) | (#4) OR (#3) OR (#2) OR (#1) | 30939 | 10:45:26 |
| 4 | "甲状腺肿物"[常用字段:智能] | 558 | 10:44:13 |
| 3 | "甲状腺占位"[常用字段:智能] | 341 | 10:43:30 |
| 2 | "甲状腺结节"[常用字段:智能] | 16992 | 10:43:04 |
| 1 | "甲状腺癌"[不加权:扩展] |  |  |
