# Supplementary material for: Diagnostic accuracy of S-Detect in distinguishing benign and malignant thyroid nodules: A meta-analysis
Source: PLoS One. 2022 Aug 5;17(8):e0272149. doi: 10.1371/journal.pone.0272149 (PMC9355179; doi:10.1371/journal.pone.0272149)
Supplement: Attachment — Submitted filename: point.docx [file pone.0272149.s006.docx]

**Reviewer #1:** The authors report a meta-analysis to evaluate the diagnostic accuracy of S-Detect in distinguishing benign and malignant thyroid nodules.

Introduction is quite general, and some statements are misleading. For example, "the sample sizes were not enough" (lines 56-57) should be better explained and referenced.

Answer:Line44-47,50-53,58-64.

Keywords. The following keywords and MeSH terms were used: ["thyroid cancer" or "thyroid neoplasm" or "thyroid tumor" or "thyroid nodule "] and [“S-Detect” or “smart detect” or “artificial Intelligence” or "computer aid diagnosis” or “machine intelligent”] (lines 60-62). Some keywords seem to be confusing. What is the meaning of "machine intelligent"? It returns only ten results in Pubmed when directly used as a keyword.

Answer:They are connected by ‘or’, which has no impact on the search results. ‘Machine intelligent’ can be deleted.

Data extraction (lines 75-78). The researchers collected the following data: the first author's surname, publication year, language of publication, study design, sample size, number of lesions, source of the subjects, "gold standard," and diagnostic accuracy.

I think the researchers collected the number of true positives, true negatives, false positives, and false negatives, instead of diagnostic accuracy. Is this correct?

Answer:Yes,you are right, i have modified it.

Furthermore, was the S-Detect version used registered? At least 2 different versions exist.

Moreover, how was the index test interpreted? S-DetectTM for Thyroid is a technology providing the features of the selected nodule with information on 6 features. Instead of directly suggesting malignancy or benignity of the nodules, the software may classify them according to three major thyroid imaging and data reporting systems: K-TIRADS, Russ, and ATA guidelines. So, did the authors collect the mode in which S-Detect was used in each study and the threshold used to "diagnose" malignant cases? Please, clarify.

Answer:We did not find the information about registration and version from these articles. I have clarified the mode.Line80-82.

I think these are important issues that should be added to the meta-regression analysis.

Why was the country of origin of the study not used as a potential variable in the meta-regression analysis?

Answer:Ok. I have added it.Table2.

Reference standard and Selection bias. The inclusion of studies with histological confirmation only is a strength (because histology is a good reference standard), but also a limitation (because surgical series are prone to selection bias). The authors should briefly discuss this issue.

Answer:Ok.line 176-178

Registration. Was this meta-analysis pre-registered in a public database? (e.g., PROSPERO). Please, clarify.

Answer:OK.Line 68-70.

There are some typos and grammar errors that should be corrected.

Answer:I have corrected them.

Reviewer #2: This meta-analysis aimed to identify the diagnostic accuracy of S-Detect 15 in distinguishing benign and malignant thyroid nodules. I have several suggestions.

1. Please provided the search strategy in each database.

Answer: I have uploaded it as supplementary materials.

2. Please provide the Fagan diagram.

Answer: Line141.Figure 8.

3. The heterogeneity within studies are very high, and please performed the subgroup analysis or meta-regression.

Answer:Table 2.

4. The PLR, NLR and DOR should be reported, too.

Answer:line134.

5 Please evaluate the threshold effect withing study.

Answer:line132.

Journal Requirements:

When submitting your revision, we need you to address these additional requirements.

1. Please ensure that your manuscript meets PLOS ONE's style requirements, including those for file naming. The PLOS ONE style templates can be found at

https://journals.plos.org/plosone/s/file?id=wjVg/PLOSOne_formatting_sample_main_body.pdf and

https://journals.plos.org/plosone/s/file?id=ba62/PLOSOne_formatting_sample_title_authors_affiliations.pdf

Answer:ok.

2. Thank you for stating the following in your Competing Interests section: "None"

Please complete your Competing Interests on the online submission form to state any Competing Interests. If you have no competing interests, please state "The authors have declared that no competing interests exist.", as detailed online in our guide for authors at http://journals.plos.org/plosone/s/submit-now

Answer:ok.

3. In your Data Availability statement, you have not specified where the minimal data set underlying the results described in your manuscript can be found. PLOS defines a study's minimal data set as the underlying data used to reach the conclusions drawn in the manuscript and any additional data required to replicate the reported study findings in their entirety. All PLOS journals require that the minimal data set be made fully available. For more information about our data policy, please see http://journals.plos.org/plosone/s/data-availability.

Upon re-submitting your revised manuscript, please upload your study’s minimal underlying data set as either Supporting Information files or to a stable, public repository and include the relevant URLs, DOIs, or accession numbers within your revised cover letter. For a list of acceptable repositories, please see http://journals.plos.org/plosone/s/data-availability#loc-recommended-repositories. Any potentially identifying patient information must be fully anonymized.

Important: If there are ethical or legal restrictions to sharing your data publicly, please explain these restrictions in detail. Please see our guidelines for more information on what we consider unacceptable restrictions to publicly sharing data: http://journals.plos.org/plosone/s/data-availability#loc-unacceptable-data-access-restrictions. Note that it is not acceptable for the authors to be the sole named individuals responsible for ensuring data access.

We will update your Data Availability statement to reflect the information you provide in your cover letter.

This information should be included in your cover letter; we will change the online submission form on your behalf.

Answer:ok.

4. Please include captions for your Supporting Information files at the end of your manuscript, and update any in-text citations to match accordingly. Please see our Supporting Information guidelines for more information: http://journals.plos.org/plosone/s/supporting-information.

Answer:ok.

5. We noticed you have some minor occurrence of overlapping text with the following previous publication(s), which needs to be addressed:

- https://medultrason.ro/medultrason/index.php/medultrason/article/view/2460

- https://www.researchsquare.com/article/rs-16146/v1

In your revision ensure you cite all your sources (including your own works), and quote or rephrase any duplicated text outside the methods section. Further consideration is dependent on these concerns being addressed.

Answer:ok. The repetition rate reduction process has been carried out.
