# Supplementary material for: Diagnostic accuracy of S-Detect in distinguishing benign and malignant thyroid nodules: A meta-analysis
Source: PLoS One. 2022 Aug 5;17(8):e0272149. doi: 10.1371/journal.pone.0272149 (PMC9355179; doi:10.1371/journal.pone.0272149)
Supplement: Attachment — Submitted filename: point-point.docx [file pone.0272149.s007.docx]

1. Search strategy. I highlighted an issue with the search criteria. While I agree that the specific keyword is not modifying the query results, the point is: are the authors sure that their strategy missed no appropriate paper? I see that the other reviewer also asked for clarification and complete queries.

The new query (“computer aid diagnosis”) is misspelled (computer-aided diagnosis being the correct spelling).

**Answer：**We are sure no appropriate paper are missed. I have corrected the query. Line 75.

2. The authors did not find the information about registration and version of the used S-Detect software. It should be discussed as a limitation of the meta-analysis.

**Answer：**Line 178.

3. The country where the study was performed should be included as a variable in the meta-regression analysis reported in Table 2, if possible. If not possible, it should at least be discussed as a limitation.

**Answer：T**he country have been included as a variable in the meta-regression analysis reported in Table 2.
